# The influence of college students’ psychological resilience on problem solving: based on eye tracking technology

**DOI:** 10.3389/fpsyg.2025.1616452

**Published:** 2025-08-15

**Authors:** Zhenguo Xu, Wenxiu Du, Huaiyan Zhao, Qing Liu, Wanli Xie

**Affiliations:** ^1^Faculty of Education, Qufu Normal University, Qufu, China; ^2^School of Communication, Qufu Normal University, Rizhao, China; ^3^Department of Tourism Information, Shandong College of Tourism and Hospitality, Jinan, China

**Keywords:** eye tracking technology, psychological resilience, problem solving, college students, cognitive processing

## Abstract

**Introduction:**

The digital intelligence era has put forward new requirements for cultivating innovative talent, and problem solving ability is one of the key abilities for the cultivation of innovative talents.

**Methods:**

Using eye tracking technology, this study explored the impact of college students’ psychological resilience on problem solving. A mixed experimental design (psychological resilience level × difficulty of questions) was employed, combining eye movement data, emotion observation record sheet, and retrospective oral reports. The differences in problem solving strategies of 24 college students in C programming were analyzed from multiple perspectives.

**Results:**

The study found that: (1) Eye movement behavior: Students with high psychological resilience exhibited longer total fixation duration, more regression counts, and greater pupil diameter changes during complex tasks. They showed comprehensive coverage and logical exploration of the stem and option areas, optimizing cognitive resource allocation through in-depth information processing. In contrast, students with low psychological resilience demonstrated shorter total fixation duration, fewer regression counts, and smaller pupil diameter changes, exhibiting “cognitive narrowing” and disordered exploration. (2) Problem solving strategies: Students with high psychological resilience employed strategies such as “segmental disassembly” and “secondary validation,” while students with low psychological resilience tended to use “random trial and error” or abandoned the task. (3) Emotion management: Students with high psychological resilience were emotionally stable, while students with low psychological resilience were prone to abandoning the task due to anxiety.

**Discussion:**

This study offers a new perspective for exploring the impact of psychological resilience of college students on problem solving and provides scientific and practical guidance for enhancing college students’ problem solving abilities.

## 1 Introduction

The era of digital intelligence puts forward new requirements for the cultivation of innovative talents, and problem solving capability is one of the key elements for cultivating innovative talents, playing a pivotal role. As future builders for the society, college students’ great problem solving capability not only helps them to cope with academic challenges, but also lays a solid foundation for their future career development. The Organization for Economic Co-operation and Development (OECD), in the OECD Learning Compass 2030, emphasized the development of key competencies for students. This is not only an important way to achieve individual happiness and collective wellbeing, but also an important safeguard for adapting to the rapid changes in the digital era. The World Development Report 2018: Learning to Realize Education’s Promise similarly pointed out the need for students to be equipped with capabilities to deal effectively with everyday tasks and challenges. In 2024, UNESCO released AI Competency Framework for Students. This is the first global scale standard document to describe student AI capabilities, which lists problem solving skills as a core literacy in the age of AI and emphasizes the development of students’ ability to critically apply AI to solve complex problems through ethical, social and technological integration. All these requirements highlight the importance of good problem solving skills.

Problem solving ability is not only influenced by intellectual factors such as basic knowledge, memory capability, and thinking ability, but also by non-intellectual factors such as interest, willpower, and beliefs (Chbeir and Carrión, 2023). Among these non-intellectual factors, psychological resilience, as a critical psychological quality for individuals to cope with stress and setbacks, plays an important role in students’ learning and life (Masten, 2014; Beese et al., 2023). It has been found that there is a significant positive correlation between students’ psychological resilience and their academic performance (Luthar et al., 2000). Psychological resilience stimulates students’ intrinsic motivation to maintain strong willpower and beliefs in the face of difficulties, which in turn improves the efficiency and quality of problem solving. Although the importance of psychological resilience has been widely recognized, the specific mechanisms by which psychological resilience affects the problem solving process remains unclear, and there is a relative lack of in-depth exploration, especially at the cognitive processing level.

Eye tracking technology, as an emerging measurement tool, is able to accurately capture an individual’s visual behavior during problem solving in real time (Seo et al., 2021; Da Silva Soares et al., 2023). This technology takes advantage of eye tracking devices to collect multidimensional visual data such as the trajectory of the individual’s eye sweep, the distribution of gaze points, the duration of gaze, and the change in pupil diameter when an individual is faced with various types of problems. By analyzing these data profoundly, the researcher can gain a deeper understanding of students’ cognitive processing during problem solving, hence providing a new perspective on the role of psychological resilience in problem solving. At the same time, the detailed data generated by eye tracking technology helps educators as well as relevant researchers to formulate effective scientific strategies. It also serves as a solid, reliable and highly convincing scientific basis for enhancing students’ problem solving skills (Rayner, 2009; Türkoğlu and Yalçınalp, 2024).

This study aims to explore the effect of college students’ psychological resilience on problem solving and to analyze it on the ground of eye tracking technology. By comparing varied eye-tracking behaviors of college students with different levels of psychological resilience in solving problems of different difficulties, the study is able to reveal the intrinsic cognitive mechanism of psychological resilience affecting problem solving, so as to provide scientific basis and practical guidance for enhancing the problem solving ability of college students.

## 2 Theoretical review

### 2.1 The concept and structural model of psychological resilience

The concept of psychological resilience was first proposed by Rutter (1979), which refers to that an individual is still capable of maintaining a well-adapted and developmental posture despite adversity. The whole concept emphasizes the dynamic process nature of psychological resilience. A bit later, Luthar et al. (2000) further interpreted the concept from an outcome-oriented perspective, pointing out that psychological resilience is the ability of an individual to positively adjust himself/herself to smoothly integrate and adapt to the surrounding environment despite of experiencing significant adversity. Heiman (2002) furthermore suggested in-depth that psychological resilience was not only power source to resist crisis and stress, but also the internal drive force for individuals to continuously grow in the face of adversity. Although the definition of psychological resilience has not yet been fully aligned in the academic circle, it is generally agreed that psychological resilience is the ability of an individual to effectively cope with and return to his or her original state, or even to achieve positive growth in the face of stress, setbacks and adversity.

In order to gain a more comprehensive understanding of the generative mechanisms of psychological resilience, scholars have constructed a variety of structural models of psychological resilience. Among them, the organizational framework of psychological resilience proposed by Mandleco and Peery (2000) inventively viewed internal and external factors as an interrelated system that worked together. This system plays a crucial role in influencing the development of psychological resilience in individuals. Kumpfer (2002) further stressed the interactions between individuals and their environments and delved into the ways in which these interactions mediated and influenced the adaptive outcomes of individuals through a range of dynamical mechanisms. In addition, Garmezy (1985) presented three different models in terms of protective and risk factors: compensatory model, protective model and preventive model. Each of these models has its own focus, but they all lay stress on the importance of the interaction between internal and external factors, and between the individual and the environment, in the formation and development of psychological resilience.

### 2.2 The concept, process, and influencing factors of problem solving

Problem solving refers to the process by which an individual, when faced with a problem situation, gradually transforms the problem from an unclear initial state to a clear and definite state through the flexible use of various cognitive operations and strategies (Newell and Simon, 1972). Schoenfeld (1985) further expressed that problem solving ability was not limited to the ability to correctly answer specific questions. It was more of a higher-order, integrative ability that encompassed the complete process and internal logic of learning to solve problems and required individuals to demonstrate deep understanding and flexible responses in the process of problem solving. However, most of the tests employed to examine students’ problem solving ability focus only on the speed and correctness of students’ problem solving, without paying attention to the process of problem solving itself. Many researchers believe that problem solving covers multiple processes (Wang and Chiew, 2010; Gunawan et al., 2018). According to Huang and Wu (2024), problem solving ability refers to a series of dynamic processes in which an individual, based on his or her existing knowledge structure and experience system, is able to keenly construct and identify the nature of a problem, and then creatively generate, effectively implement, and scientifically evaluate the best solution. The British psychologist Wallas (1926) raised a four-stage model of problem solving, which are the preparation period, the gestation period, the clarification period and the validation period.

The multi-stage of the problem solving process leads to the fact that many factors can have impacts on problem solving (Aydın Kılıç and Ercoşkun, 2024). Shin et al. (2025) found through their study that metacognitive scaffolding as an effective learning strategy significantly optimizes an individual’s cognitive load and improves problem solving skills in collaborative programming. By distributing questionnaires to 156 college students and conducting statistical analyses, Gönderen Çakmak and Ayhan BaŞer (2024) revealed that an evidence-based practice course was beneficial for developing lifelong learning skills and problem solving abilities. Chi et al. (1981) discovered through their study that there was a significant difference between experts and novices in problem solving, with experts possessing greater domain knowledge and being able to more accurately characterize problems and select effective strategies. Tian et al. (2018) spotted that metacognitive knowledge had a direct effect on the mathematical problem solving process through the questionnaire method. Individuals’ awareness of their self-cognitive abilities directly affects the efficiency and accuracy of their problem solving. Apart from cognitive factors, motivational and emotional factors should not be ignored. Positive motivation can stimulate individuals’ intrinsic motivation, prompting them to concentrate more on problem solving activities. Emotional arousal refers to the physiological activation level from calmness to excitement. Wundt (1907) divided emotions into three dimensions: pleasure, excitement, and tension. Izard (2004) believed that emotions had four dimensions: pleasure, excitement, tension, and conviction. Russell and Barrett (1999) divided emotions into valence and arousal. These divisions indicate that arousal is an essential component of emotions. Emotional arousal refers to the degree of activation of an organism’s emotional state, which provides a preparatory state for subsequent behavior and also has a certain impact. Many previous studies have focused on the dimension of emotional valence, but as research deepens and expands, researchers are increasingly paying attention to the important role of emotional arousal. Stefanucci and Storbeck (2009) demonstrated through research that emotional arousal can regulate a person’s perception of height. Moderate emotional arousal maintains individuals’ attention and effort, ensuring that they remain calm and productive in the face of challenges (Chen et al., 2021). Moderate emotions refer to individuals being in the optimal range of emotional arousal during a task, manifested as mild activation of the sympathetic nervous system physiologically, subjective experience of tension but controllable psychologically, and effort investment that matches task needs in behavior (Hamm et al., 2002; Levenson, 2002). This state can maintain concentration and avoid cognitive resource depletion caused by excessive arousal. However, negative emotions such as anxiety and frustration can be disruptive to cognitive processing and impede problem solving (Eysenck et al., 2007). According to emotion regulation theory and dynamic systems theory, emotions are not independent of cognitive interference variables, but rather synergistic agents that interact dynamically with cognitive resources. The way individuals regulate emotions directly affects the functional direction of emotions (Gross, 1998). Zheng et al. (2024) measured the facial expressions and galvanic skin data of 47 medical students through an intelligent tutoring system and detected that stable emotions were more likely to lead to problem solving. From the perspective of specific questions, this study holds the opinion that problem solving refers to the thinking and cognitive processes exhibited by individuals as they progressively advance a problem from an initial state to a goal state.

### 2.3 The application of eye tracking technology in problem solving research

Eye tracking technology is one of the emerging technologies widely used in the research field of education science to analyze the learning process of students (Seo et al., 2021; Da Silva Soares et al., 2023). Based on the principle that eye movements are closely related to cognitive processing, eye tracking technology reveals the cognitive processing mechanisms of an individual during problem solving by recording eye movement metrics such as gaze point, gaze duration, scanning path, and pupil diameter (Türkoğlu and Yalçınalp, 2024). Exploring learners’ cognitive processes based on eye tracking technology has become an prominent way of research in education and psychology (Xue and Zhu, 2024). Zang et al. (2022) identified a high correlation between students’ eye-movement behavior and performance on a science problem solving assessment. Uygun et al. (2022) examined and reported on students’ geometric misunderstandings in detail from different perspectives with the help of data relevant to eye-tracking. Their study found out that students’ eye movement trajectories tend to reflect the difficulties and cognitive conflicts they encounter when faced with geometric misunderstanding items. Using eye tracking technology, Andrzejewska et al. (2015) detected that using the formal notation of a programming language to represent algorithms had difficulties in solving simple tasks and that eye tracking technology could optimize the process of programming learning. Ahn et al. (2024) focused on the effect of problem presentation on students’ problem solving process. Using eye tracking technology, they compared students’ attention allocation between two different presentations: comics and text, and discovered that comics, with their intuitive and vivid characteristics, are more capable of attracting students’ attention than traditional text presentations, thus potentially facilitating their problem solving skills. What’s more, Tóthová et al. (2021) achieved a qualitative analysis of high school students’ ability to use the Periodic Table of Elements for problem solving tasks through eye tracking technology. Their study not only revealed students’ cognitive strategies in using the Periodic Table, but also provided valuable feedback and suggestions for chemistry teaching. In all, the eye tracking technology provides intuitive and accurate data support for in-depth understanding of information acquisition, attentional allocation, and cognitive load changes during problem solving, and is conducive to reveal differences in problem solving across individuals.

### 2.4 The effect of psychological resilience on problem solving

Psychological resilience refers to the ability to actively recover and adapt in adversity, and is a key element of emotional and cognitive abilities required for students to cope with inherent challenges in education (Geldenhuys et al., 2014). Therefore, psychological resilience has a significant effect on problem solving. Individuals with higher psychological resilience are more inclined to adopt coping strategies that are productive and positive when faced with problems. They are able to maintain calmness and clarity of mind under stress, manage and overcome negative emotions effectively, and thus are able to focus their attention more intently, analyze problems in depth, and generate effective solutions creatively (Romano et al., 2021; Puente-Hidalgo et al., 2024). Galve-González et al. (2025) noticed that students with high psychological resilience were able to view stress as an opportunity and challenge for growth and proactively seek and find solutions to problems, whereas students with low psychological resilience might be more prone to anxiety and helplessness in the face of stress, which affected their problem solving skills. St Clair-Thompson et al. (2014) categorized psychological resilience into four components: commitment, challenge, control and assertiveness, and proclaimed that there was a significant correlation between psychological resilience and students’ grades as well as attendance through questionnaire method. Shao et al. (2025) announced that psychological resilience mediated the relationship between teacher support and commitment to learning, and that individuals with high psychological resilience were better able to adapt to challenges and difficulties encountered during the learning process, leading to increased engagement and problem solving. Liu and Sun (2019) pointed out that psychological resilience positively predicts psychological resource investment, that is, individuals with high psychological resilience are more inclined to actively allocate cognitive resources in stressful situations. Wen (2024) found that total fixation duration can reflect the depth of subjects’ processing of picture information, and the longer the total fixation duration, the easier it is to conduct deep cognitive processing. However, current researches on psychological resilience influencing problem solving, especially in conjunction with emerging research methods such as eye tracking technology, should be further explored, which provides an important research direction for this study.

### 2.5 The present study

**FIGURE 1:**
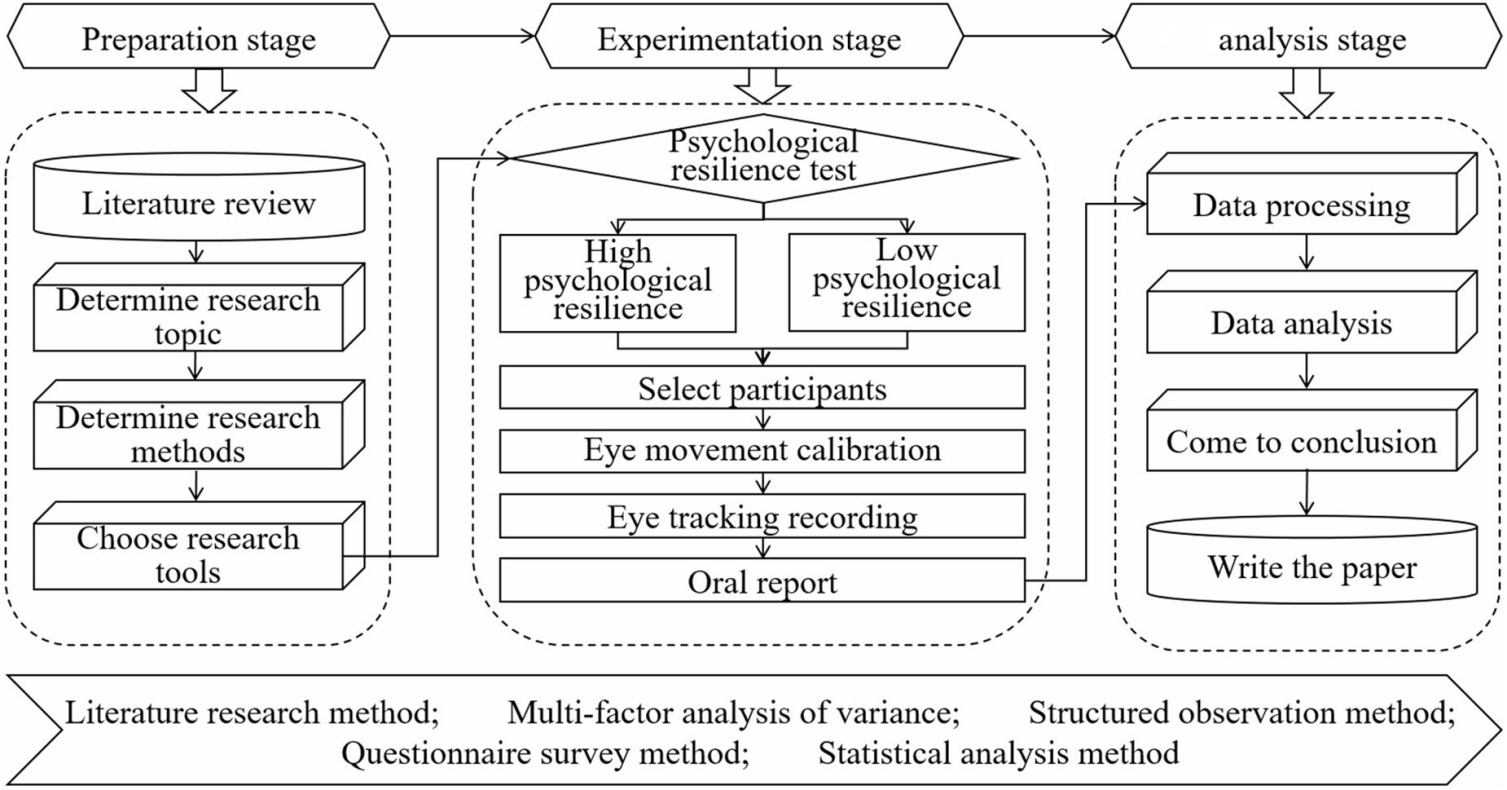
Research procedure diagram.

Existing research mainly explores the relationship between psychological resilience and problem solving through questionnaire surveys, which have unique value in capturing individual self-perception experiences. However, the questionnaire survey method has significant limitations, such as strong subjectivity and susceptibility to perfunctory or false responses. Based on this, this study introduces eye tracking technology as a supplementary means to compare the eye movement behavior of college students with different levels of psychological resilience when dealing with different difficult tasks, revealing the impact of psychological resilience on the internal cognitive process of problem solving from a behavioral perspective. Based on this, this study introduces eye tracking technology as a supplementary means, and uses a mixed experimental design of 2 (high/low psychological resilience level) × 3 (difficulty of questions: simple/medium/complex) to compare the eye movement behavior of college students with different levels of psychological resilience when dealing with different difficult tasks, revealing the impact of psychological resilience on the internal cognitive process of problem solving. Focus on addressing the following issues: Are there any differences in eye movement behavior among college students with different levels of psychological resilience when solving problems? Are there any differences in problem solving strategies among college students with different levels of psychological resilience? Are there any differences in cognitive processing efficiency among college students with different levels of psychological resilience when solving problems? This study believes that the combination of eye tracking data and questionnaire survey results can provide complementary evidence for a comprehensive and in-depth understanding of the complex relationship between psychological resilience and problem solving, and thus provide more scientific and rich theoretical support and practical guidance for improving college students’ problem solving abilities. The research procedure of this study is shown in Figure 1.

## 3 Materials and methods

### 3.1 Experimental design

Firstly, the psychological resilience scale was used to measure the level of psychological resilience of college students, and the psychological resilience of college students was classified into low and high levels based on the average of test scores. The psychological resilience scale is mainly divided into three dimensions: resilience, strength, and optimism. Secondly, C programming test questions were drafted, and the test questions were classified into simple difficulty, medium difficulty and complex difficulty according to the difficulty coefficient of the test questions. The C language programming test questions will be selected from the 2024 National Computer Rank Examination (Level 2 C Language) real questions. Then, the C programming test questions were used as eye movement test materials, and the eye tracking technology was used to conduct a 2 (psychological resilience) × 3 (test difficulty) two factor mixed experiment on college students, and the emotional state of students when solving the questions was recorded through structured observation method. Finally, after the experiment, experimental subjects’ problem solving thinking processes were collected and analyzed through retrospective oral reports.

### 3.2 Experimental subjects

First, a paper version of the psychological resilience questionnaire was distributed through a simple random sampling method in the second year of a university in Shandong Province. For the convenience of statistical analysis, the paper version of the questionnaire was collected and then data were manually entered into Excel spreadsheets. The statistical results showed that 963 questionnaires were distributed and 959 questionnaires were recovered, with a recovery rate of 99.6%, of which 947 were valid questionnaires, with an effective rate of 98.7%. The age of the experimental subjects ranged from 19 to 23 years old. The average age was 21.4 ± 1.3 years old. The maximum age was 23 years old. The minimum age was 19 years old. In terms of gender distribution, there were 633 female students, accounting for 66.8%, and 314 male students, accounting for 33.2%. Based on the average of the test scores, psychological resilience is classified into low level (≤65 points) and high level (>65 points). Among them, there were 454 students with low level of psychological resilience, accounting for 47.9%. There were 493 students with high level of psychological resilience, accounting for 52.1%. Secondly, this study conducted prior power analysis to determine the appropriate sample size. G*Power indicates that in order to obtain sufficient power to detect larger effect sizes (Cohen’s d = 0.8, α = 0.05, statistical power 1-β = 0.8), the required sample size for each group is n = 12 (total sample size 24), which is sufficient for the planned analysis (calculation formula:$n=2\times \frac{{\left(Z_{\alpha /2}+Z_{\beta }\right)}^{2}}{d^{2}}$, where Z_0.025_ = 1.96, Z_0.2_ = 0.84, substituting d = 0.8), n≈12.3, Round to 12). In the sophomore year of the university, 6 boys and 6 girls were selected from the low level and high level of psychological resilience students respectively by stratified random sampling, and a total of 24 college students participated in the eye tracking experiment. All experimental subjects had uncorrected or corrected visual acuity of 1.0 or above, with no visual problems such as astigmatism. In addition, all experimental subjects had completed the C Programming course and scored at the upper intermediate level on the final exam, with similar levels of knowledge, which as a result effectively controlled prior experience interference. This study was approved by the Ethics Committee of Qufu Normal University, and all experimental subjects signed informed consent forms. The basic information of the research subjects is shown in Table 1.

**TABLE 1:** Basic information of research subjects.

	Questionnaire					Eye movement experiment				
Research subjects	Number of people	Gender		Age		Number of people	Gender		Age	
	N	Male	Female	M	SD	N	Male	Female	M	SD
High psychological resilience	493	164	329	21.4	1.5	12	6	6	21.2	2.6
Low psychological resilience	454	150	304	21.4	1.2	12	6	6	21.2	2.3

### 3.3 Experimental materials

#### 3.3.1 Psychological resilience scale

The classical psychological resilience scale was used in this study. The classical psychological resilience scale was developed by Connor and Davidson (2003) and revised into the Chinese version of the Psychological Resilience Inventory Scale by Yu and Zhang (2007). The scale contains three dimensions: resilience, strength and optimism. Resilience refers to the ability to be calm, determined, responsive, and in control in the face of challenges. Strength refers to the ability to not only recover from setbacks, but also to develop and grow. Optimism refers to having confidence in overcoming adversity and seeing things in a positive light. The scale consists of 25 items and is rated on a 5-point Likert scale, with higher scores indicating higher levels of psychological resilience. Reliability and validity analysis was conducted on the scale, and it was found that the internal consistency of the total scale and its three sub dimensions was good. The Cronbach’s alpha coefficients were 0.91, 0.89, 0.91, and 0.88, respectively, indicating good reliability of the scale. Confirmatory factor analysis showed that the model fit was ideal (χ^2^/df = 1.928, RMSEA = 0.049, GFI = 0.954, CFI = 0.928, NFI = 0.924, TLI = 0.956), and the scale structure validity was good.

#### 3.3.2 C programming test questions

In this study, the 2024 National Computer Grade Examination (Level 2 C) questions were selected as experimental test materials. This specific exam is sponsored by the Examination Center of the Ministry of Education of China and is categorized as the national standardized exam, and its real questions are authoritative and widely recognized due to strict drafting of questions and rigid scoring standards and great specifications. The employing of standardized test materials helps to reduce the errors caused by the differences in materials, ensure the stability and accuracy of the experimental results, and thus enhance the credibility of the study.

The process of preparing test questions is meticulous and rigorous. Firstly, 12 questions from the 2024 National Computer Grade Examination (Level 2 C) questions were screened as the initial test questions by experienced front-line university teachers. These questions covered the knowledge scope including macro definitions, pointers, arrays, functions, selection structures, and loop structures. Then 963 test questions were distributed to sophomores and 960 were recovered, with a recovery rate of 99.7%. After excluding invalid responses such as incomplete responses and regular responses, the final number of valid responses was 909, with a validity rate of 94.7%. Quantitative analysis was performed using Excel 2021 software to obtain the difficulty of each question. The difficulty of the test questions was expressed as a difficulty coefficient. The difficulty coefficient was calculated by the score rate (Crocker and Algina, 1986). The specific formula is *P* = R/N (R represents the number of students who passed the questions and N represents the total number of participants in the test). The results of the difficulty coefficient analysis are shown in Table 2. Generally, *P* < 0.4 is considered as a complex problem, 0.4 ≤ *P* < 0.7 as a medium problem and *P* ≥ 0.7 as a simple problem. Finally, two expert teachers were invited to evaluate and screen out 2 questions from each pool of simple, medium and complex difficulty, totaling 6 questions as the final test material for the experiment.

**TABLE 2:** Results of analyzing the difficulty of test questions.

Item number	1	2	3	4	5	6
Difficulty coefficient	0.83	0.85	0.58	0.52	0.25	0.64
Difficulty level	Simple	Simple	Medium	Medium	Complex	Medium
**Item number**	**7**	**8**	**9**	**10**	**11**	**12**
Difficulty coefficient	0.72	0.81	0.62	0.38	0.29	0.75
Difficulty level	Simple	Simple	Medium	Complex	Complex	Simple

#### 3.3.3 Student emotion observation record form

In this study, the direct observation method was used to conduct the research work, and a structured observation record form was designed to record in detail the emotional performance of students during the problem solving process. This study is based on the basic theory of emotions and further expands the types of emotions, dividing them into nine categories: focus, distraction, happiness, sadness, anger, anxiety, panic, disgust, and surprise. Among them, focus and distraction reflect task oriented cognitive engagement, while anxiety and panic are different intensity manifestations of fear. During the experiment, this study observed individuals’ facial expressions in detail, such as the curvature of the corners of the mouth, the shape of the eyebrows, and changes in eye contact, as important criteria for determining emotional types. Based on the above research methods and criteria, this study developed a set of student emotion observation record form, aiming to achieve accurate recording and in-depth analysis of various types of emotions mentioned above. Through pre experimental observation, it was found that the participants’ emotion observation record form in the task were highly consistent with their behavioral performance. At the same time, this study invited three experts in the field of emotional psychology to evaluate the correlation between indicators and emotional behavior in problem solving, as well as the comprehensiveness and discrimination of indicators. Experts used a rating scale of 1–5 points (1 point represents completely unrelated, 5 points represents highly related), and the results showed that all indicators scored ≥ 4 points, indicating that the tool has good reliability.

#### 3.3.4 Oral reports

In order to ensure the objectivity and accuracy of the experimental process, this study used retrospective oral reports to facilitate in the validation analysis. Retrospective oral reports are the process of having subjects verbally describe and report their mental activities after completing a certain kind of assignment (Dong, 2004). This study focuses on the following three stages based on the stage division theory of problem solving thinking process proposed by Wu et al. (2006). (1) Question reading stage: evaluate students’ ability to accurately capture key information and clearly define research questions. (2) Problem solving stage: examine students’ ability to quickly apply relevant knowledge, effectively choose problem solving strategies, construct well-organized problem solving plans, and successfully complete test question solutions. (3) Inspection stage: analyze whether students have the initiatives and awareness to actively check the correctness of problem solving answers and can quickly take corrective measures for errors found.

### 3.4 Experimental instruments and environment

This study utilized the aSee A3 eye tracker to collect eye movement data from each experimental subject. The data acquisition frequency of this instrument is 60 Hz, with an accuracy of 0.3°. Simultaneously, a 15.6-inch laptop with computer monitor screen resolution of 1,920 × 1,080 pixels and a screen refresh rate of 165 Hz was equipped to acquire the feature of high precision and reliability for the experiment. The experimental materials were displayed on the screen, and during the problem solving process, the eye tracker would track and record the movement trajectory of the eyes in real time. Additionally, the study also used aSee Studio software synchronized with eye tracking to collect data. In order to ensure the accuracy of the results, the experiment was conducted in a quiet, constant-temperature smart classroom with appropriate lighting conditions to minimize external interference. Testees sat on soft chairs, required to keeping their eyes at a distance of about 50 cm from the equipment and keeping their eyes sights on the screen at all times to improve the efficiency and effectiveness of eye movement data collection.

### 3.5 Experimental procedure

The experiment was conducted in a smart classroom of a university in Shandong Province and was collaboratively completed by two experimenters. The two experimenters were completely unaware of the grouping of participants’ psychological resilience. Both of them are familiar with basic emotion theory, have the ability to accurately identify micro expressions, and are proficient in the operation process and recording standards of structured observation method. To ensure reliability, they practiced by repeatedly watching video cases and ultimately achieved the evaluation standard of Cohen’s Kappa > 0.7 for inter rater consistency. The explanation for Cohen’s Kappa value is as follows: the range of 0.61–0.80 is defined as “strong consistency” (McHugh, 2012), and Cohen’s Kappa > 0.7 indicates that the scoring results of the two main participants have high reliability. Cohen’s Kappa is calculated using the formula *k* = (Po-Pe)/(1-Pe), where Po is the observed consistency rate and Pe is the random consistency rate. Before the experiment began, experimenter A first briefly introduced the experimental procedure and related precautions to all subjects, then guided them to complete the 9 point calibration of the eye tracker and officially conducted the test. During the testing process, experimenter A needs to continuously observe changes in students’ emotions and fill out the student emotion observation record form. At the same time, experimenter B synchronously performed emotion observation and recording tasks, and conducted oral interviews with all subjects in the later stage of the experiment, requiring them to briefly narrate their problem solving process and record their oral expressions in detail. All experimental subjects participated in the experiment after signing the informed consent form.

### 3.6 Data processing

The aSee Studio software accompanying the aSee A3 eye tracker was used to record eye-tracking data, and the raw data were cleaned and organized to remove invalid data and outliers to ensure data quality. All data were output in the format of an Excel file and data were analyzed in SPSS 27 software. Apart from that, eye movement hotspot maps and eye movement trajectory maps were output as JPG images.

## 4 Results

In order to explore the differences in problem solving among students with different levels of psychological resilience, this study selected five eye movement indicators: total fixation duration, regression count, pupil diameter change rate, eye movement hotspot map, and eye movement trajectory map, and combined with the emotion observation record form and retrospective oral reports for comprehensive analysis.

### 4.1 Total fixation duration

Total fixation duration is the sum of the durations of all the fixations during the problem solving process, which is a key indicator of the overall level of cognitive processing input of the subjects during the problem solving process (Chen and Zheng, 2014). The data of total fixation duration were first tested for normal distribution. In this study, Shapiro-Wilk method and skewness coefficient and kurtosis coefficient test were used to test the normal distribution of total fixation duration. When the significance level is bigger than 0.05, it indicates that the data follows a normal distribution; When the absolute value of skewness is less than 1.5 and the absolute value of kurtosis is less than 1.5, it follows a normal distribution (Tabachnick and Fidell, 2019). The results showed that the significance was greater than 0.5 and the absolute values of skewness and kurtosis were both less than 1.5, indicating that the data followed a normal distribution. Secondly, Levene’s test for homogeneity of variances was conducted on the total fixation duration, and the results showed that the data met the homogeneity of variance (*p* > 0.05).

Based on this, this study further conducted a multivariate analysis of variance on the total fixation duration of college students with different levels of psychological resilience when facing different difficulty problems. The Mauchly method was used to test the assumption of sphericity, and the within-subject variable difficulty of questions obeyed the assumption of sphericity (*p* > 0.05). The results of the multifactor ANOVA showed that the main effect of the within-subjects variable difficulty of questions was highly significant, *F*(2, 44) = 140.199, *p* < 0.001, η^2^ = 0.864. The main effect of the between-subjects variable psychological resilience level was highly significant, *F*(1, 22) = 200.744, *p* < 0.001, η^2^ = 0.901. The interaction effect was highly significant, *F*(2, 44) = 44.471, *p* < 0.001, η^2^ = 0.669, and thus required a simple effects analysis. The results showed that there was a significant difference between the two levels of psychological resilience at the three difficulties of questions (*p* < 0.001). Comparison of means revealed that on simple problems, students with high psychological resilience had an average total fixation duration of 13.514 s more than students with low psychological resilience. On medium problems, students with high psychological resilience had an average total fixation duration of 18.815 s more than students with low psychological resilience. On complex problems, students with high psychological resilience had an average total fixation duration of 52.047 s more than students with low psychological resilience. The higher the difficulty of questions, the greater the difference in total fixation duration between the two psychological resilience students. Among low level of psychological resilience students, there are significant differences in total fixation duration between simple and medium problems, and between simple and complex problems. Among the students with high level of psychological resilience, there are significant differences in the difficulty of the three test questions. The total fixation duration of students with high levels of psychological resilience gradually increased with the difficulty of the questions. In contrast, students with low levels of psychological resilience showed different characteristics when solving problems: when facing simple problems, their total fixation duration was the shortest, only 17.568 s; when dealing with complex problems, it was the second shortest, 36.223 s; and when solving medium difficult problems, the total fixation duration was instead the longest, 40.854 s. The results of the simple effects analysis are shown in Table 3.

**TABLE 3:** Results of simple effects analysis (total fixation duration).

Difficulty of questions	Level of psychological resilience		I-J	SE	Sig^b^	95% CI^b^	
	(I)M_*Low*_	(J)M_*High*_				Lower-bound	Upper-bound
Simple	17.568	31.082	−13.514*	1.977	<0.001	−17.614	−9.414
Medium	40.854	59.669	−18.815*	3.319	<0.001	−25.699	−11.931
Complex	36.223	86.27	−52.047*	4.063	<0.001	−60.474	−43.62
**Level of psychological resilience**	**Difficulty of questions**		**I-J**	**SE**	**Sig^b^**	**95% CI^b^**	
	**(I)M**	**(J)M**				**Lower-bound**	**Upper-bound**
Low	Simple	Medium	−23.286*	2.391	<0.001	−29.481	−17.091
	Simple	Complex	−18.655*	3.511	<0.001	−25.753	−7.557
	Medium	Complex	4.631	3.374	0.186	−2.111	15.373
High	Simple	Medium	−28.587*	2.391	<0.001	−34.782	−22.392
	Simple	Complex	−55.188*	3.511	<0.001	−64.286	−46.09
	Medium	Complex	−26.601*	3.374	<0.001	−35.343	−17.859

*Indicates that the significance level of the mean value difference is less than 0.05.

### 4.2 Regression count

Regression counts refer to the number of times a subject returns from the current gaze point to content that has already been gazed at. The number of regression counts indicates that the subject has doubts about the previously processed information or needs to reconfirm it. The regression counts were first subjected to a normal distribution test and Levene’s test for homogeneity of variances. The results showed that the data were normally distributed (*p* > 0.05) and satisfied variance alignment (*p* > 0.05).

Based on this, a multifactor ANOVA was conducted to analyze the regression counts of college students with different levels of psychological resilience when facing different difficulty problems. Mauchly’s method was used to test the assumption of sphericity, and the within-subjects variable difficulty of questions obeyed the assumption of sphericity (*p* > 0.05). The results of the multifactor ANOVA showed that the main effect of the within-subjects variable difficulty of questions was highly significant*, F*(2, 44) = 187.724, *p* < 0.001, η^2^ = 0.895. The main effect of the between-subjects variable level of psychological resilience was highly significant, *F*(1, 22) = 245.837, *p* < 0.001, η^2^ = 0.918. The interaction effect between the two was highly significant, *F*(2, 44) = 102.908, *p* < 0.001, η^2^ = 0.824, thus requiring a simple effects analysis. The results showed that there was a significant difference between the two levels of psychological resilience at the three difficulties of questions (*p* < 0.001). Comparison of means revealed that on simple problems, students with high psychological resilience had an average of 0.834 regression counts higher than students with low psychological resilience. On medium problems, students with high psychological resilience had an average of 1.833 higher regression counts than students with low psychological resilience. On complex problems, students with high psychological resilience had an average of 5.083 higher regression counts than students with low psychological resilience. The higher the difficulty of questions, the greater the difference in the number of regression counts between the two types of psychological resilience students. At the same time, there was a significant difference in the difficulty of questions between the two of the three tests. The number of regression counts for students with high levels of psychological resilience tended to increase with the difficulty of the questions. On the contrary, students with low levels of psychological resilience showed different characteristics when solving the questions: when facing simple questions, the average number of regression counts was only 0.333; when dealing with complex problems the number of regression counts rose to 1.167; and when coping with medium difficult questions, the number of regression counts reached 1.750 instead. The results of the simple effects analysis are shown in Table 4.

**TABLE 4:** Results of simple effects analysis (regression counts).

Difficulty of questions	Level of psychological resilience		I-J	SE	Sig^b^	95% CI^b^	
	(I)M_*Low*_	(J)M_*High*_				Lower-bound	Upper-bound
Simple	0.333	1.167	−0.834**	0.181	<0.001	−1.209	−0.458
Medium	1.750	3.583	−1.833*	0.291	<0.001	−2.437	−1.23
Complex	1.167	6.250	−5.083*	0.245	<0.001	−5.591	−4.575
**Level of psychological resilience**	**Difficulty of questions**		**I-J**	**SE**	**Sig^b^**	**95% CI^b^**	
	**(I)M**	**(J)M**				**Lower-bound**	**Upper-bound**
Low	Simple	Medium	−1.417*	0.229	<0.001	−2.01	−0.824
	Simple	Complex	−0.834*	0.218	0.003	−1.399	−0.268
	Medium	Complex	0.583*	0.209	0.032	0.041	1.126
High	Simple	Medium	−2.416*	0.229	<0.001	−3.01	−1.824
	Simple	Complex	−5.083*	0.218	<0.001	−5.649	−4.518
	Medium	Complex	−2.667*	0.209	<0.001	−3.209	−2.124

*Indicates that the significance level of the mean value difference is less than 0.05.

### 4.3 Pupil diameter change rate

Pupil diameter, as an important physiological indicator, can effectively reflect the cognitive and emotional state of students in the process of problem solving. The degree of pupil diameter change is significantly and positively correlated with the degree of cognitive engagement (Kahneman, 1973). Pupil diameter change rate refers to the dynamic magnitude of change in pupil diameter relative to the baseline value during the performance of a task by an individual. Baseline Pupil Diameter is the size of an individual’s pupil diameter in the resting state, without cognitive or emotional load. The pupil diameter change rate is calculated as: the pupil diameter change rate = $\left(\frac{D_{\text{task}}-D_{\text{baseline}}}{D_{\text{baseline}}}\right)\times 100\%$, where *D*_*task*_ is the mean pupil diameter during the task and *D*_*baseline*_ is the baseline value. The pupil diameter change rate was first subjected to the normal distribution test (*p* > 0.05) and Levene’s test for homogeneity of variances. The results showed that the data were normally distributed and satisfied variance alignment (*p* > 0.05).

Based on this, a multifactor ANOVA was conducted to analyze the pupil diameter change rate of college students with different levels of psychological resilience when facing different difficulty problems. The Mauchly method was used to test the assumption of sphericity, and the within-subject variable difficulty of questions obeyed the assumption of sphericity (*p* > 0.05). The results of the multifactor ANOVA showed that the main effect of the within-subjects variable difficulty of questions was highly significant, *F*(2, 44) = 79.833, *p* < 0.001, η^2^ = 0.784. The main effect of the between-subjects variable level of psychological resilience was significant, *F*(1, 22) = 11.527, *p* = 0.003, η^2^ = 0.344. The interaction effect was highly significant, *F*(2, 44) = 13.790, *p* < 0.001, η^2^ = 0.385. Therefore a simple effects analysis was required. The results showed that there was a significant difference in pupil diameter change rate between students with high psychological resilience and those with low psychological resilience on complex problems (*p* < 0.001), and there was no significant difference on both medium and complex difficulty problems (*p* > 0.05). Comparison of means revealed that there was almost no difference between students with high psychological resilience and those with low psychological resilience on simple and medium problems, which were only 0.50% and 1.47% higher. In contrast, on complex problems, the pupil diameter change rate was 9.3% higher for students with high psychological resilience than for students with low psychological resilience. Among the students with low level of psychological resilience, there are significant differences in pupil diameter change rate between simple and medium problems, and between simple and complex problems. Among the students with high level of psychological resilience, there are significant differences in the difficulty of the three test questions. The pupil diameter change rate of students with high level of psychological resilience showed a significant increase with increasing difficulty of the questions. However, students with low levels of psychological resilience showed differential cognitive load characteristics during problem solving: when facing simple problems, the pupil diameter change rate was only 11.404%, which was the lowest level of difficulty among the three groups. The rate of change rose to 17.988% when dealing with complex questions. Instead, the rate of change jumped to 19.675% when dealing with medium questions. The results of the simple effects analysis are shown in Table 5.

**TABLE 5:** Results of simple effects analysis (pupil diameter change rate).

Difficulty of questions	Level of psychological resilience		I-J	SE	Sig^b^	95% CI^b^	
	(I)M_*Low*_	(J)M_*High*_				Lower-bound	Upper-bound
Simple	11.404%	11.901%	−0.50%	1.039	0.637	−2.652	1.659
Medium	19.675%	21.141%	−1.47%	1.942	0.458	−5.493	2.561
Complex	17.988%	27.288%	−9.30%*	1.482	0 < 0.001	−12.372	−6.226
**Level of psychological resilience**	**Difficulty of questions**		**I-J**	**SE**	**Sig^b^**	**95% CI^b^**	
	**(I)M**	**(J)M**				**Lower-bound**	**Upper-bound**
Low	Simple	Medium	−8.27%*	1.554	< 0.001	−12.298	−4.244
	Simple	Complex	−6.58%*	1.18	< 0.001	−9.642	−3.526
	Medium	Complex	1.69%	1.123	0.442	−1.222	4.596
High	Simple	Medium	−9.24%*	1.554	< 0.001	−13.267	−5.213
	Simple	Complex	−15.39%*	1.18	< 0.001	−18.445	−12.329
	Medium	Complex	−6.15%*	1.123	< 0.001	−9.056	−3.238

*Indicates that the significance level of the mean value difference is less than 0.05.

### 4.4 Eye movement hotspot map

The eye movement hotspot map is a visualization tool that intuitively shows the distribution and pattern of subjects’ attention. It uses color and density differences to vividly indicate the overlapping areas of visual attention and their degree of attraction for the subjects. Specifically, the longer the gaze duration, the darker the color and higher the density, reflecting a higher level of attention to that area. In this study, questions 1, 4, and 5 were selected to represent three different levels of difficulty: simple, medium, and complex, respectively, with the aim of exploring in depth the differences exhibited by students with different levels of psychological resilience in problem solving.

**FIGURE 2:**
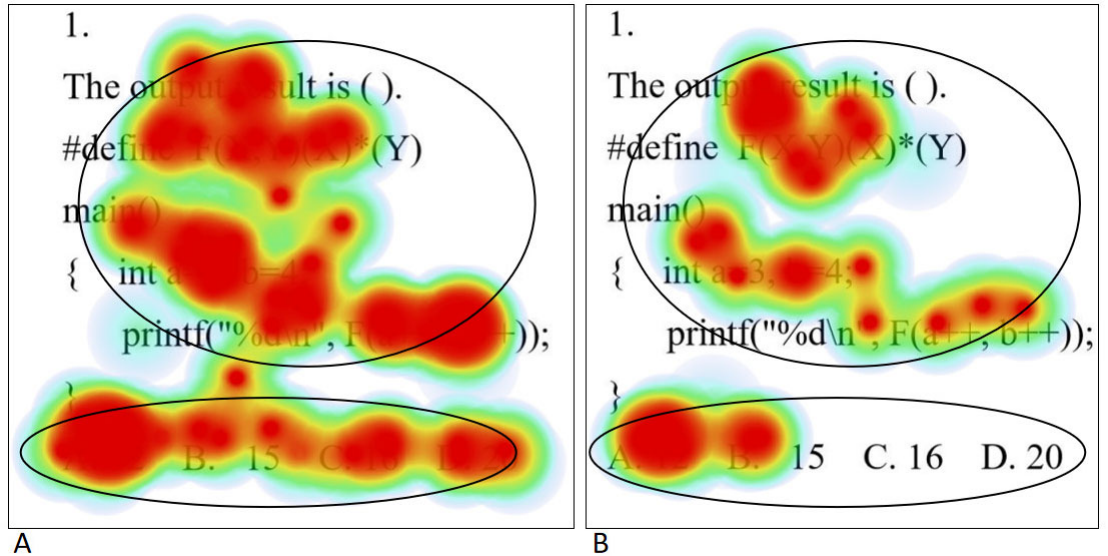
Simple questions eye movement hotspot maps [high level of psychological resilience **(A)** and low level of psychological resilience **(B)**].

The eye movement hotspot maps of college students with high level of psychological resilience (A) and low level of psychological resilience (B) when solving simple problems are shown in Figure 2. Overall, students with high levels of psychological resilience showed a more comprehensive and intensive distribution of gaze. In the stem region, students with high level of psychological resilience generally focus on all parts of the stem, whereas students with low level of psychological resilience tend to concentrate on key statements in the stem. In the option area, students with high level of psychological resilience examine all options thoroughly, whereas students with low level of psychological resilience tend to focus only on the correct option. In addition, when solving simple problems, high level psychological resilience students had significantly higher gaze densities than low level psychological resilience students, which is highly consistent with the results of the statistical analysis of total fixation duration. In order to realize scientific quantitative comparative analysis, this study divided the AOIs of the question stem part and the option part, and used the total fixation duration and the fixation counts as the quantitative indexes of the eye movement hotspot map. Total fixation duration refers to the sum of the duration of all fixations within each AOI, which measures the overall cognitive processing input of the subjects during problem solving. Fixation count refers to the total number of fixations within each AOI. The higher the number of fixations, the darker the color of the region in the hotspot map. Independent samples *t*-tests were conducted on them separately, and the results showed that the total fixation duration and the fixation counts in the question stem and option areas of the high level psychological resilience group were significantly higher than those of the low level psychological resilience group (all *p* < 0.05). The results are shown in Table 6.

**TABLE 6:** Analysis of differences in eye movement indicators between different psychological resilience groups.

Difficulty of questions	Indicator	Region	High psychological resilience(M ± SD)	Low psychological resilience(M ± SD)	*p*	Cohen’s d
Simple	Total fixation duration	Question stem region	20.1 ± 4.3	12.7 ± 2.4	*p* < 0.05	2.13
		Option region	4.3 ± 2.7	2.2 ± 1.1	*p* < 0.05	1.02
	Fixation count	Question stem region	12.4 ± 3.2	6.1 ± 0.3	*p* < 0.05	2.77
		Option region	4.1 ± 1.1	1.9 ± 0.5	*p* < 0.05	2.57
	Mean saccade distance	Question stem region	140 ± 20	155 ± 22	*p* < 0.05	−0.71
		Option region	120 ± 15	115 ± 13	*p* > 0.05	0.36
	Fixation sequence entropy	Question stem region	0.8 ± 0.2	0.9 ± 0.3	*p* > 0.05	−0.39
		Option region	1.0 ± 0.2	1.1 ± 0.3	*p* > 0.05	−0.39
Medium	Total fixation duration	Question stem region	45.5 ± 9.8	34.2 ± 3.3	*p* < 0.05	1.55
		Option region	5.5 ± 2.4	2.7 ± 1.5	*p* < 0.05	1.40
	Fixation count	Question stem region	16.1 ± 3.3	8.9 ± 0.9	*p* < 0.05	2.98
		Option region	4.9 ± 1.3	3.2 ± 1.4	*p* < 0.05	1.26
	Mean saccade distance	Question stem region	144 ± 15	149 ± 17	p > 0.05	−0.31
		Option region	122 ± 14	140 ± 22	*p* < 0.05	−0.98
	Fixation sequence entropy	Question stem region	1.1 ± 0.3	1.8 ± 0.4	*p* < 0.05	−1.98
		Option region	1.4 ± 0.3	2.0 ± 0.4	*p* < 0.05	−1.70
Complex	Total fixation duration	Question stem region	68.3 ± 9.2	30.8 ± 4.6	*p* < 0.05	5.16
		Option region	6.9 ± 1.7	3.5 ± 1.4	*p* < 0.05	2.18
	Fixation count	Question stem region	21.3 ± 4.5	10.1 ± 3.9	*p* < 0.05	2.66
		Option region	6.4 ± 1.3	2.3 ± 1.6	*p* < 0.05	2.81
	Mean saccade distance	Question stem region	115 ± 12	210 ± 30	*p* < 0.05	−4.16
		Option region	111 ± 13	149 ± 27	*p* < 0.05	−1.79
	Fixation sequence entropy	Question stem region	1.3 ± 0.3	2.5 ± 0.5	*p* < 0.05	−2.91
		Option region	1.6 ± 0.4	2.8 ± 0.6	*p* < 0.05	−2.35

**FIGURE 3:**
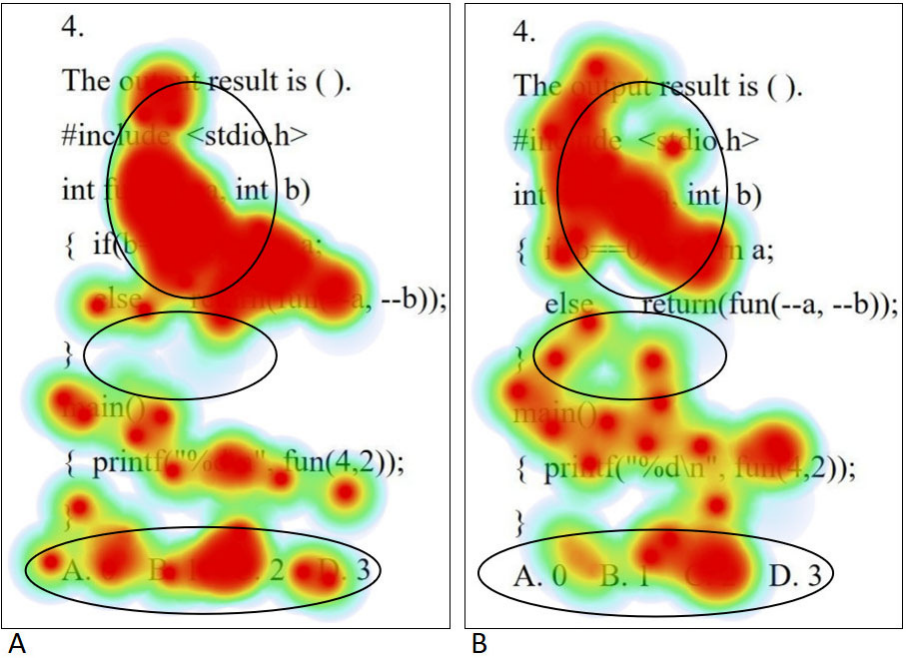
Medium questions eye movement hotspot maps [high level of psychological resilience **(A)** and low level of psychological resilience **(B)**].

When solving medium problems, the eye movement hotspot maps of college students with high level of psychological resilience (A) and low level of psychological resilience (B) are shown in Figure 3. On the whole, students with high level of psychological resilience mainly focused on the question itself, while students with low level of psychological resilience not only distributed their focus on the question, but also scattered to areas unrelated to the question. Specifically, in the stem region, students with high level of psychological resilience were able to achieve full attention coverage of all the statements, showing in-depth grasp of the information of the question; on the contrary, students with low level of psychological resilience were prone to deviate from the stem and shift their attention to areas unrelated to the question, showing the characteristics of distraction. In the option area, students with high levels of psychological resilience tended to examine all options comprehensively, in contrast to students with low levels of psychological resilience who only focused on the first three options, that is, only focused on the correct option. This finding is highly consistent with the results of the statistical analysis of total fixation duration. Independent samples *t*-tests on the total fixation duration and fixation counts of students with different psychological resilience at medium difficulty levels were conducted separately, and the results showed that the total fixation duration and the fixation counts of the high level psychological resilience group were significantly higher than those of the low level psychological resilience group in the question stem and option areas (all *p* < 0.05). The results are shown in Table 6.

**FIGURE 4:**
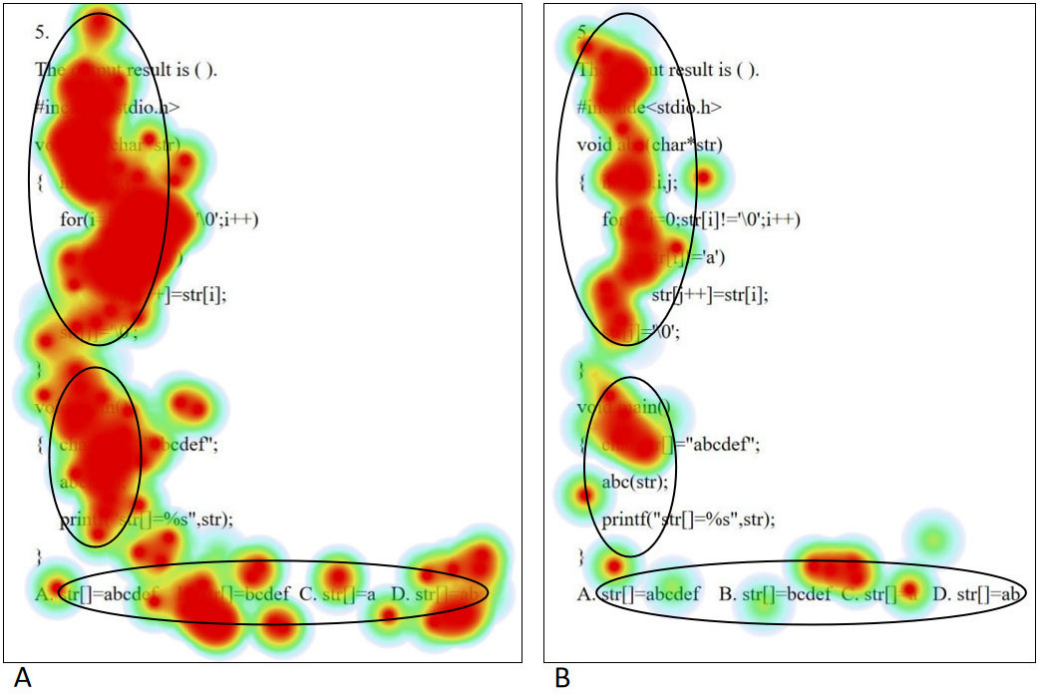
Complex questions eye movement hotspot maps [high level of psychological resilience **(A)** and low level of psychological resilience **(B)**].

The eye movement hotspot maps of college students with high level of psychological resilience (A) and low level of psychological resilience (B) when solving complex problems are shown in Figure 4. Overall, the distribution of gaze points was more comprehensive and dense for the high level psychological resilience students. Specifically, in the first half of the stem, both groups demonstrated gaze coverage of the entire utterance, but the low level psychological resilience students had a lower density of gaze than the high level psychological resilience students, whereas in the second half of the stem, the low level psychological resilience students had a significantly lower number of gaze points. In the option area, students with high level of psychological resilience demonstrated careful reading of all options, whereas students with low level of psychological resilience showed a significant gaze deficit. When solving complex problems, high level psychological resilience students had a significantly higher range and density of gaze points than low level psychological resilience students, and this difference was most pronounced across the three problem categories, which is highly consistent with the results of the statistical analysis of total fixation duration. Independent samples *t*-tests on the total fixation duration and fixation counts of students with different psychological resilience at complex difficulty were conducted separately, and the results showed that the total fixation duration and fixation counts of the high level psychological resilience group were significantly higher than those of the low level psychological resilience group in the question stem and option areas (all *p* < 0.05). The results are shown in Table 6.

### 4.5 Eye movement trajectory map

The eye movement trajectory map visualizes the movement path of the line of sight between gaze points, including the order of the gaze points, the duration of the gaze, and the distance between the gaze points, by superimposing the eye movement information with a specific interface image. In this study, the eye movement trajectories of college students with different psychological resilience were analyzed for three types of problems: simple, medium and complex. To ensure the consistency of the study, eye movement trajectory maps matching the eye movement hotspot maps were selected for in-depth analysis.

**FIGURE 5:**
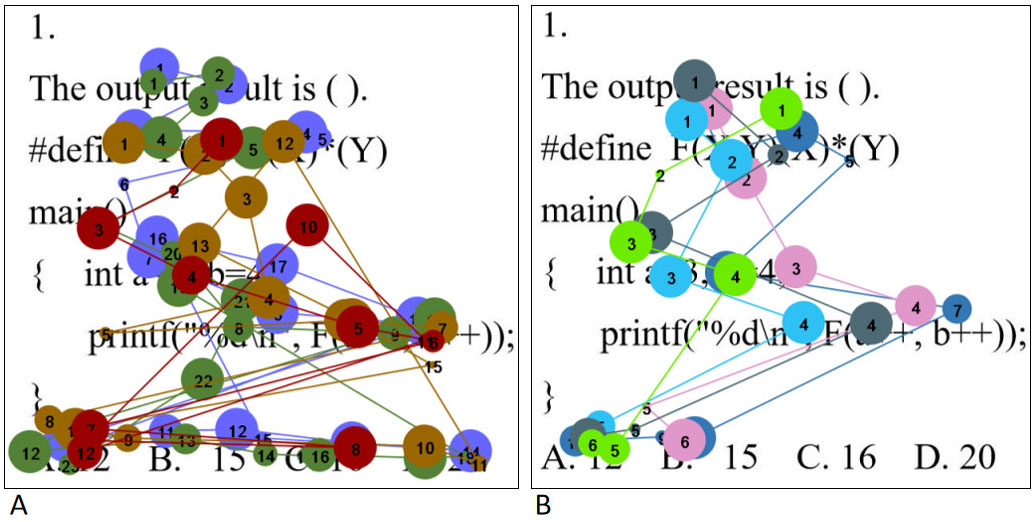
Simple problem eye movement trajectory maps [high level of psychological resilience **(A)** and low level of psychological resilience **(B)**].


When solving simple problems, the eye movement trajectory maps of high level of psychological resilience (A) and low level of psychological resilience (B) college students are shown in Figure 5. Students with high level of psychological resilience exhibit longer gaze paths in their eye-movement trajectories when faced with simple problems, indicating that they engage in more comprehensive thinking during information processing. At the same time, they have a significantly higher number of gaze points, which implies that they tend to scrutinize every detail more meticulously when comprehending problems and sifting through information. In addition, students with high level of psychological resilience also showed higher regression counts, meaning that they review key points after working on a question to ensure the correct answer this. In contrast, students with low level of psychological resilience had relatively short eye movement trajectories when solving simple problems, showing their limitations in observation. The smaller number of gaze points reflects that they may lack sufficient care and patience in information processing. Meanwhile, students with low levels of psychological resilience have almost no retrospective behavior, which may imply that they are overconfident in solving problems and lack the necessary reflection and validation, thus increasing the risk of errors. The above differences in eye movement characteristics were highly consistent with the results of the analysis of regression counts. In order to realize scientific quantitative comparative analysis, this study adopted mean saccade distance and fixation sequence entropy as the quantitative indexes of eye movement trajectory map. Mean saccade distance reflects the average movement of subjects from one gaze point to the next in a visual task. Fixation sequence entropy refers to the probability distribution of the transfer of gaze points among different AOIs, which measures the “disorder degree” of the sequence, the larger the entropy, the more random the sequence is. Fixation sequence entropy needs to be calculated. First, the basic data were obtained: ➀ gaze point coordinates (x, y pixel coordinates): the position of each gaze point on the screen was recorded; ➁ gaze point timestamp: the start/end time of each gaze point was recorded (accurate to milliseconds); ➂ stimulus material information: the visual stimulus presented to the subjects should be aligned with the coordinate system of the eye movement data. Second, the stimulus material was divided into two AOIs (Question stem region; Option region). Then the preprocessed gaze point coordinates were mapped to the AOIs to obtain a chronological AOI sequence. Finally, the “state transfer probability matrix” was calculated based on the AOI transfer sequence, and then substituted into the entropy formula.

Transfer⁢probability⁢formula:

$$P\left(i\to j\right)=\frac{\mathrm{Number}\,\mathrm{of}\,\mathrm{transfers}}{\mathrm{Total}\,\mathrm{number}\,\mathrm{of}\,\mathrm{transfers}}$$

Entropy⁢calculation⁢formula:

$$H=-\sum_{i=1}^{n}\sum_{j=1}^{n}P\left(i\to j\right)\cdot {\text{log}}_{2}P\left(i\to j\right)$$

(*n* is the total number of AOIs, and the unit of the H is “bit”.)

Independent samples *t*-tests were conducted on them separately, and the results showed that there was no significant difference in fixation sequence entropy between the high level psychological resilience group and the low level psychological resilience group in the question stem and option areas (all *p* > 0.05), and only in the question stem portion of the high level psychological resilience group, the mean saccade distance was significantly smaller than that of the low level psychological resilience group (*p* < 0.05). The results are shown in Table 6.

**FIGURE 6:**
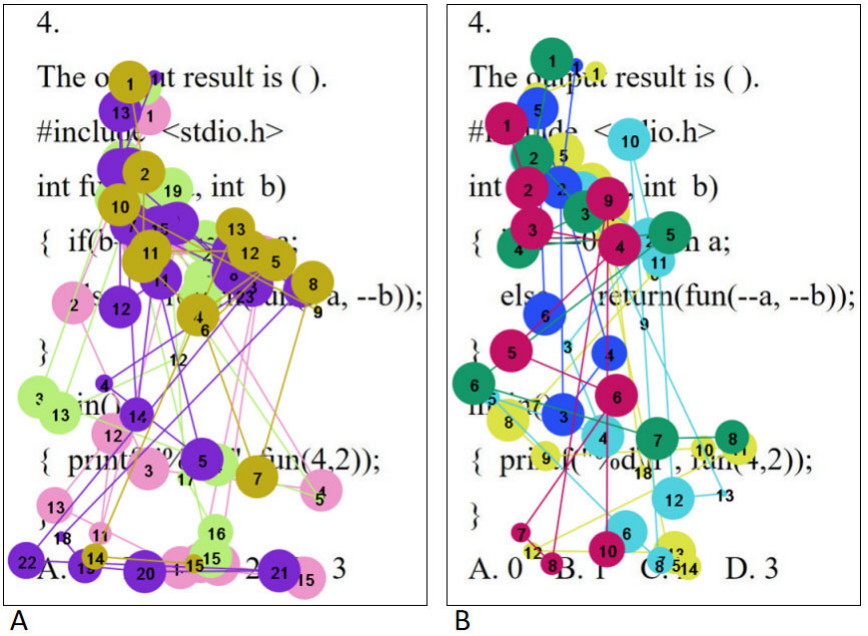
Medium problem eye movement trajectory maps [high level of psychological resilience **(A)** and low level of psychological resilience **(B)**].

The eye movement trajectory maps of high level of psychological resilience (A) and low level of psychological resilience (B) college students when solving medium problems are shown in Figure 6. The total length of gaze paths was similar for students with high and low levels of psychological resilience. Analyzing the eye movement trajectories in depth, both high level psychological resilience and low level psychological resilience students showed some retrospective behaviors during the problem solving process; however, the two groups of students were quite different in the specific manifestations of their eye movement trajectories. Students with high level of psychological resilience showed an orderly jumping of their eye-movement trajectories between key statements, and this jumping seemed to follow some kind of internal logic or problem solving strategy, indicating that they were more efficient and precise in information processing. In contrast, the eye-movement trajectories of students with low level of psychological resilience appeared to jump in a chaotic and large-amplitude manner, lacking apparent logic and direction. This disorganized eye movement pattern may reflect their difficulties in information integration, problem comprehension, or deficiencies in attention allocation, resulting in difficulties in forming a coherent thought path during problem solving. Independent samples *t*-tests of mean saccade distance and fixation sequence entropy for students with different psychological resilience at medium difficulty levels, respectively. The results showed that the high level psychological resilience group had a significantly lower fixation sequence entropy in the question stem and option areas than the low level psychological resilience group (both *p* < 0.05), and the mean saccade distance was significantly smaller in the option section than in the low level psychological resilience group (*p* < 0.05). The results are shown in Table 6.

**FIGURE 7:**
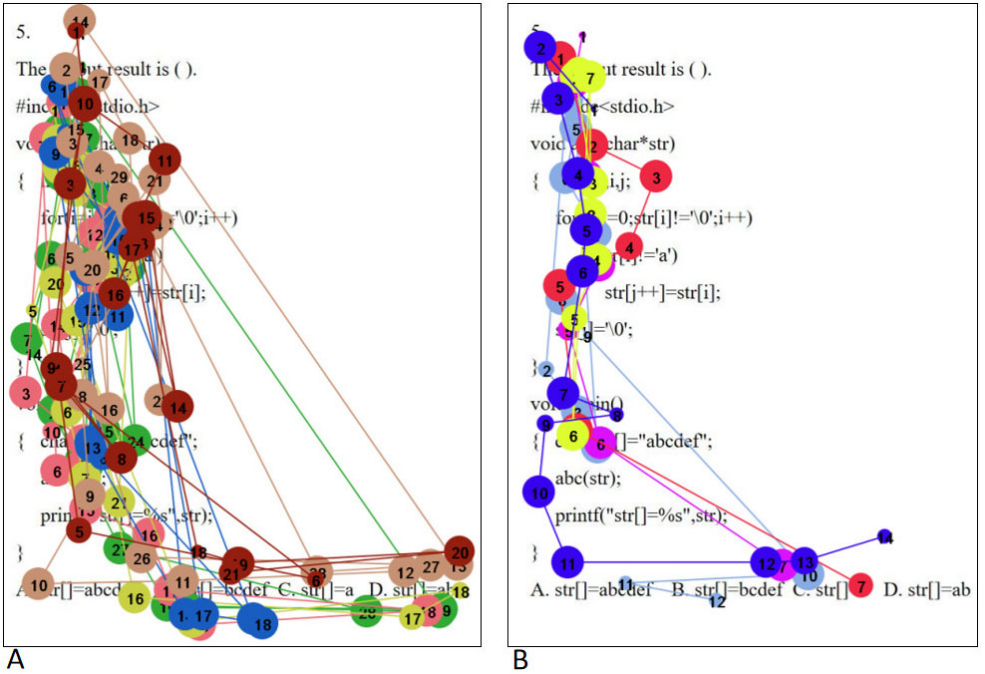
Complex problem eye movement trajectory maps [high level of psychological resilience **(A)** and low level of psychological resilience **(B)**].

The eye movement trajectory maps of high level of psychological resilience (A) and low level of psychological resilience (B) college students when solving complex problems are shown in Figure 7. Students with high level of psychological resilience had significantly more total gaze path lengths than those with low level of psychological resilience, suggesting that they invested more cognitive resources in problem solving and engaged in more comprehensive and in-depth visual exploration. Also, their total fixation duration was significantly longer than that of students with low levels of psychological resilience, reflecting that they were more careful and patient in information processing. Further analysis revealed that students with high level of psychological resilience looked back at the question several times when facing complex problems, and this kind of retrospective behavior helped them understand the question’s intention more accurately, integrate the information, and form a more complete strategy for solving the problem. On the contrary, students with low level of psychological resilience tended to show an avoidance attitude when facing difficult problems, no longer looking back at the problem, or even giving up the problem after reading only half of the problem, which is obviously not conducive to effective problem solving. It is worth noting that the differences in eye movement trajectories between high level psychological resilience and low level psychological resilience college students showed a high degree of consistency with their performance in total fixation duration and regression counts. Independent samples *t*-tests were conducted on mean saccade distance and fixation sequence entropy of students with different psychological resilience at complex difficulty, respectively. The results showed that the mean saccade distance and fixation sequence entropy of the high level psychological resilience group were significantly lower than those of the low level psychological resilience group in the question stem and option regions (all *p* < 0.05). The results are shown in Table 6.

### 4.6 Student emotion observation record form

This study used a structured observation method to record the emotional changes of experimental subjects during the process of completing different difficulty test questions through the student emotion observation record form. The results showed that students with high level of psychological resilience remained focused and emotionally stable throughout the process of completing the questions, with no significant emotion swings. In contrast, students with low level of psychological resilience showed more complex emotional performance when facing different difficulties of questions. In addition to the state of concentration when working on simple problems, they showed emotions of happiness and surprise, which may be related to the sense of relief and accomplishment brought by the simplicity of the problems. When doing medium difficulty of questions, they show some degree of distraction, anxiety and panic, which suggests that the medium difficulty of questions is beginning to cause them some stress and challenge. When doing complex problems, their emotional performance was more negative, with a variety of emotions such as distraction, sadness, anger, anxiety and panic, which reflected their anxiety and powerlessness in the face of challenges. The specific performance of students’ emotions is shown in Table 7. The data in the table represent the average number of emotion appearances per person per question.

**TABLE 7:** Statistical table of student emotion observation.

Difficulty of questions		Simple		Medium		Complex	
Level of psychological resilience		High	Low	High	Low	High	Low
Emotional classification	Focus	1	1	1	1	1	1
	Distraction	0	0	0	0.625	0	0.25
	Happiness	0	0.875	0	0	0	0
	Sadness	0	0	0	0	0	0.625
	Anger	0	0	0	0	0	0.5
	Anxiety	0	0	0	0.625	0	1
	Panic	0	0	0	0.5	0	0.5
	Disgust	0	0	0	0	0	0
	Surprise	0	0.375	0	0	0	0

### 4.7 Retrospective oral reports

This study analyzed the retrospective oral reports of problem solving process of college students with different psychological resilience for three types of problems: simple, medium and complex. To ensure the consistency of the study, the retrospective oral reports matching the eye movement hotspot map were selected for thorough analysis in this study. The retrospective oral reports are shown in Table 8.

**TABLE 8:** Retrospective oral reports.

Difficulty of questions	Level of psychological resilience	Report content
Simple	High	(1) “When I reviewed the question, I focused on macro definitions and self-incrementing symbols. a++ is post-incrementing, an operator that first returns the current value of the variable a before increasing the value of a by 1. So the question went straight to 3 × 4 = 12, which gave me 12. I checked again to make sure the answer was correct.” (2) “I’ve done this kind of question but I rechecked it just in case.”
	Low	(1) “I was very happy to see this question, which is very simple, a++ for post-incremental self-incrementation is a direct 3 × 4, so I chose option A straight away.” (2) “I’ve done similar problems with macro definitions before, and I was very excited to see that I immediately chose the answer.”
Medium	High	(1) “I saw the question, read through the question carefully and focused on the key statements, thought carefully and compared the answers over and over again, and I found the right answer.” (2) “This question is a bit of a challenge, but I’m sure I can do it.”
	Low	(1) “I saw the question, got a little anxious, looked at the key statements and options, and picked an answer that was probably correct.” (2) “I scanned the question, saw that the question tested recursive functions, and chose the answer directly.”
Complex	High	(1) “While working on the question, I could clearly feel that the question was difficult, but I believed that I could do it, I read the question as a whole first, and then I used the strategy of comprehending the statements in segments, and finally did it, and then after that, I examined each of the options to determine the correct answer.” (2) “This question was difficult, I first identified the key statements, while later determining what the program does, and finally brought all the options into the program one by one and then found the correct answer.”
	Low	(1) “I was a little overwhelmed and very anxious when I saw the codes. The questions were not read through and I just gave up.” (2) “I’ve never gotten this kind of puzzle right before, and I picked a random answer based on luck.”

Through in-depth analysis of the oral reports, the following findings can be summarized:

1. During the reading stage, students with low level of psychological resilience, when faced with complex problems, often chose to give up their answers right in the process of reviewing the questions and lacked the willingness to think deeply and persist.
2. During the problem solving stage, students with high psychological resilience showed strong knowledge transfer ability and emotional stability, and the problem solving steps were clear and organized; while students with low psychological resilience showed chaotic problem solving steps and were prone to fall into emotional fluctuations of complacency or nervousness and anxiety.
3. During the checking stage, students with high psychological resilience levels would verify the solution results twice to ensure the accuracy of the answers. In contrast, students with low level of psychological resilience generally lacked awareness of checking their answers and were prone to overlook potential errors.

## 5 Discussion

### 5.1 The effect of psychological resilience on cognitive processing efficiency

As an important psychological trait, psychological resilience plays a key regulatory role in cognitive processing, and it significantly enhances the cognitive processing efficiency of college students in problem solving situations by finely optimizing the allocation and dynamic regulation of cognitive resources (Sun et al., 2022). The findings suggest that students with high level of psychological resilience exhibit longer total fixation duration as well as higher regression counts when confronted with complex problems. At the depth level of cognitive processing, high psychological resilience students are able to more fully explore the potential information in the question and construct more complete and accurate cognitive representations by extending gaze time and increasing regression counts (Puente-Hidalgo et al., 2024). Based on the resource allocation theory, high psychological resilience individuals possess superior ability to allocate attentional resources (Wickens, 2008). They are able to flexibly adjust the direction and intensity of the allocation of attentional resources according to task demands and cognitive load. In complex tasks, high level psychological resilience students actively prolong their gaze on the details of the question and give more cognitive attention to the key information to ensure an accurate understanding of the question content. At the same time, the acquired information was verified and corrected through repeated retrospective behaviors to improve the accuracy and reliability of the information. This result further confirms that high psychological resilience students achieve efficient use of cognitive resources during cognitive processing through the consistent performance of behavioral strategies and eye movement characteristics. In addition, as an important physiological indicator reflecting cognitive load and attentional regulation, the dynamic change of pupil diameter can intuitively reveal an individual’s physiological state and cognitive effort during cognitive processing (Kahneman, 1973). The present study found that the pupil diameter change rate of high level psychological resilience students in complex tasks reached 27.288%, which was much higher than that of low level psychological resilience students, which was 17.988%. This data comparison clearly indicates that high level psychological resilience students are able to actively cope with the increase in cognitive load by proactively regulating their attentional resources when facing complex tasks. They were not only able to withstand higher cognitive loads, but also able to flexibly adjust their cognitive strategies to adapt to the task demands, and this ability of active regulation is an important manifestation of psychological resilience at the cognitive level (Beese et al., 2023). This is consistent with the results of previous studies (Ou-Yang and Li, 2021; Sun et al., 2022).

### 5.2 The effect of psychological resilience on visual attention allocation

The spatial allocation pattern of visual attention is an intuitive manifestation of the role of psychological resilience in problem solving, which is manifested in the differences between the comprehensiveness of the distribution of gaze points and the logic of trajectories (Ou-Yang and Li, 2021; Sun et al., 2022). The level of psychological resilience significantly influences the distribution pattern and integration logic of visual attention. The eye movement hotspot maps of high psychological resilience students showed a more comprehensive gaze coverage of the stem and option regions, especially in the second half of the complex problem where high density of gaze remained. This behavior suggests that high level psychological resilience individuals are able to reinforce the completeness of question representations through sustained information retrieval (Puente-Hidalgo et al., 2024). In contrast, low psychological resilience students showed significant cognitive narrowing: focusing only on the key words of the question stem in simple tasks and abandoning the reading of the second half of the question stem early in complex tasks. In terms of the dynamic characterization of attentional trajectories, students with high level of psychological resilience demonstrated strategy-oriented trajectories in problem solving of medium and complex problems, such as linear scanning from stem to options or cyclic validation between key statements, reflecting their implicit knowledge of problem structure (Newell and Simon, 1972; Romano et al., 2021). The entropy value of the fixation sequence is relatively small. In contrast, the trajectories of students with low levels of psychological resilience show disorganized random jumps, such as frequent switching of gaze points but lack of logical correlations, which may lead to fragmentation of information integration (Lee et al., 2020). The entropy value of the fixation sequence is relatively high. Differences in gaze point distribution and gaze trajectories among students with different psychological resilience suggest that psychological resilience can optimize the efficiency of visual exploration by enhancing cognitive flexibility (Masten, 2014). This reveals the central role of psychological resilience in reconciling breadth and depth of attention, that is, balancing the tension between information coverage and depth of processing by dynamically adjusting gaze strategies (Bi and Reid, 2017).

### 5.3 The effect of psychological resilience on problem solving strategies

High or low psychological resilience determines the quality of problem solving strategy selection, execution, and monitoring, a difference that was fully revealed through multidimensional cross-validation of oral reports and eye-movement data (Shao et al., 2025; Chvátal et al., 2024). During the reading phase, high psychological resilience students demonstrated explicit global reading strategies; while low psychological resilience students relied on intuitive quick scanning. The differentiation was even more pronounced during the problem solving stage: faced with complex code questions, high level psychological resilience students used a segmented disassembly strategy, confirming the logical connections between modules through high-frequency retrospective behavior. Low level psychological resilience students fell into a random trial-and-error mode due to intimidation, and eye-movement trajectories showed disorganized jumps between code segments and options without a complete understanding of the code logic (Zheng et al., 2024; Shao et al., 2025). Differences in metacognitive monitoring abilities were further emphasized during the checking phase. High level psychological resilience students proactively implemented a secondary validation strategy, as evidenced by a significant increase in the number of regression counts and total fixation duration during this phase. Low level psychological resilience students generally lacked awareness of checking and had no subsequent validation sessions due to premature abandonment in complex tasks. The underlying mechanism for this strategy difference fits highly with Wallas’s (1926) four-stage model of problem solving. High level psychological resilience students accumulate structured information during the preparation phase (comprehensive problem review), form hypotheses through logical gaze trajectories during the gestation phase (information integration), pinpoint solution paths during the clarification phase (strategy generation), and ultimately utilize metacognitive monitoring during the validation phase (answer checking) (Flavell, 1979). In contrast, low level psychological resilience students’ problem solving strategies are characterized by shallow processing or strategy abandonment, and the severance of their eye-movement behavior from problem solving efficacy confirms the central role of psychological resilience in optimizing the dynamic adaptation of strategies through enhanced cognitive flexibility (Masten, 2014; Mansoor et al., 2023).

### 5.4 The effect of psychological resilience on emotion management ability

Emotional management plays an important role in problem-solving, and this mechanism has been systematically verified through a triple chain of evidence of structured emotion observation record form, oral reports and eye movement behaviors. The structured emotion observation record form showed that students with low psychological resilience showed much higher frequency and intensity of anxiety and panic symptoms such as frowning and frequent blinking than students with high psychological resilience in both medium and complex tasks, and many of them gave up their answers due to emotional breakdowns in the complex tasks, revealing that emotional dysregulation directly undermines task persistence (Liu et al., 2024). Oral reports further elucidated the mechanisms of the emotion-cognition interaction: low level of psychological resilience students reported that they were “at a loss and very anxious when solving complex problems.” In contrast, high level psychological resilience students maintain stable emotions through positive self-suggestion, which keeps visual attention focused on the problem solving itself (Garcia-Blanc et al., 2023). The interaction effect of pupil diameter change rate [*F*(2, 44) = 13.790, *p* < 0.001, η^2^ = 0.385] revealed key differences. High level psychological resilience students were in the optimal activation zone for emotional arousal in complex tasks, and their pupils dilated to both enhance visual sensitivity and avoid cognitive overload, consistent with Kahneman (1973) cognitive load theory. However, students with low level of psychological resilience were caught in the “low arousal-low processing” state due to emotional loss, and their pupil diameter change rate was much smaller than that of students with high levels of psychological resilience, confirming their psychological avoidance of complex tasks. This is consistent with the research results of Liu Z. et al. (2025). This divergence stems from differences in cognitive reappraisal strategies (Gross, 1998): high level of psychological resilience reconstructed stress as a growth opportunity and maintained motivation to solve the problem through meaning shifts, as evidenced by “secondary validation” behaviors during the checking phase and sustained gaze during the second half of the question. Students with low level of psychological resilience viewed stress as a threat, triggering the “fight or flight” response, which resulted in a vicious cycle of anxiety-inefficiency-failure by tilting cognitive resources toward emotion rather than problem solving. Liu L. et al. (2025) also reached consistent findings. This mechanism supports Masten (2014) “stress-growth” model, which suggests that psychological resilience not only buffers against emotional disturbances, but also enhances persistence in problem solving by stimulating goal-directed behaviors, leading to a virtuous circle of “challenge-strategy-success.”

## 6 Conclusion

Based on eye tracking technology, this study systematically explored the effects of college students’ psychological resilience on problem solving. The following main conclusions were drawn. First, psychological resilience significantly affects cognitive processing efficiency. High psychological resilience students face complex problems with longer total fixation duration and more regression counts. They can deeply process key information and have a higher pupil diameter change rate, demonstrating stronger ability to deploy attentional resources. Second, psychological resilience influences visual attention distribution patterns. Students with high psychological resilience have a more comprehensive distribution of attention points, especially in complex problems to maintain a high density of attention to the details of the question stem, and the trajectory of attention is logical, while students with low psychological resilience showed disorderly jumping in the fixation track. Third, psychological resilience affects problem solving strategies. Students with high psychological resilience adopt global strategies during the reading stage, use segmental disassembly and other methods during the problem solving stage, and adopt secondary verification strategies during the checking stage. Low psychological resilience students intuitively scan and lack awareness of checking. Fourth, psychological resilience affects problem solving through emotion. Students with high psychological resilience are emotionally stable and can focus on the problem itself, while students with low psychological resilience are prone to negative emotions such as anxiety and panic in medium and complex problems, leading to distraction and even abandonment. In summary, psychological resilience affects college students’ problem solving by influencing cognitive processing efficiency, visual attention allocation mode, problem solving strategy and emotion management ability. This provides a scientific basis for improving college students’ problem solving ability.

### 6.1 Contribution and implications

Theoretically, this study enriches the application of psychological resilience theory in the field of higher education. The mechanism of psychological resilience in the problem solving process was revealed through eye tracking technology, which provided a new perspective for the understanding of cognitive processing. Methodologically, this study inventively used eye tracking technology to provide a new method of data collection for psychological and educational research, which promotes the wide application of this technology in related fields. In the practical regard, the research results can provide a useful reference for college educators, help them improve students’ psychological resilience through targeted mental health education, optimize the training path of problem solving strategies, and enhance students’ problem solving ability in the face of academic problems.

### 6.2 Limitations and future research

Firstly, the sample size of this study is relatively small and only suitable for detecting large effects (*d* ≥ 0.8). Based on this, future research should strive to expand the sample size and expand the scope of the study to more regions and different types of schools, in order to enhance the representativeness and reliability of the study.

Second, the study was relatively homogeneous, solely focusing only on programming problem solving processes. To be more comprehensive and academically in-depth, future studies should consider including diverse task types, such as mathematical problems, logical reasoning tasks, or creative problem solving.

Thirdly, there are limitations in the research methodology. This study uses the average score as the entry point for psychological resilience grouping, which has certain limitations. In the future, more refined grouping strategies such as extreme value method will be adopted to make the research more rigorous. Meanwhile, the retrospective oral reporting method used has the following limitations: firstly, memory bias may affect data reliability. Participants’ memories of past decisions are easily disrupted by time intervals, which may result in blurring or selective memory, leading to the loss of key cognitive details; Secondly, the tendency toward rationalization afterwards may distort the actual decision-making process. Individuals may unconsciously construct a “reasonable” explanation for their behavior when reviewing, masking their true motives or conflicting psychology at the time; Thirdly, there is a lag in capturing real-time decisions. Retrospective reports rely on post hoc reconstruction, making it difficult to accurately reproduce the cognitive dynamics at the moment of decision-making; Fourthly, the results are easily constrained by the participants’ language expression and reflective abilities. Some participants may have insufficient language organization skills or limited depth of reflection, resulting in information omissions or expression deviations in the report content. Future research can optimize from the following aspects: firstly, combining real-time recording tools to shorten the recall interval and reduce the impact of memory decay; Secondly, a multi-source data cross validation mechanism is introduced to combine oral reports with objective data such as physiological indicators, reducing post rationalization bias; Again, attempt to design a hybrid approach that synchronously uses the “Think Loud Protocol” to record real-time cognitive processes at critical decision-making stages, in order to compensate for the lag of retrospective methods; Finally, in response to language proficiency differences, visual aids can be used to guide participants to supplement their descriptions and improve data integrity.

## Data Availability

The original contributions presented in this study are included in this article/supplementary material, further inquiries can be directed to the corresponding author.
